# Minimally invasive discectomy versus microdiscectomy/ discectomy for symptomatic lumbar disc herniation

**Published:** 2012-11

**Authors:** Vafa Rahimi-Movaghar, Mohammad Rasouli, Farhad Shokraneh, Maziyar Moradi-lakeh, Alex Vakaro, Mohsen Sadeghi-Naini

**Affiliations:** ^a^Deparment of Neurosurgery - Sina Hospital, Tehran University of Medical Sciences, Tehran, Iran.; ^*b*^Research Centre for Neural Repair, University of Tehran, Tehran, Iran.; ^*c*^Rothman Institute of Orthopaedics, Thomas Jefferson University, Philadelphia, PA, US.; ^*d*^Medical Librarianship and Information Sciences, Tabriz University of Medical Sciences, Tabriz, Iran.; ^*e*^Tehran University of Medical Sciences, Tehran, Iran.; ^*f*^Department of Orthopaedic Surgery and Neurosurgery,Thomas Jefferson University Hospital, The Rothman Institute.

## Abstract

**Background:**

Lumbar discectomy is a surgery to remove all or part of a disc cushion that helps protect the spinal column. These cushions, called disks, separate the spinal vertebrae/bones. When one of the disks herniates (moves out of place) in patients with protruded disc, the soft gel inside pushes through the wall of the disk. The disk may then place pressure on the spinal cord and nerves that are coming out of the spinal column.

The lumbar discectomy procedure remained basically unchanged until the operating microscope enhanced the visualization of the operative field in 1978. This new operation was recognized as lumbar microdiscectomy because it was performed through a smaller incision, with less dissection than standard open lumbar discectomy. Microdiscectomy is regarded generally as a technical modification of standard discectomy, rather than a separate procedure. In a systematic review by Gibson and Waddell, results of microdiscectomy for treatment of lumbar disc prolapse was “broadly comparable” to the standard open lumbar discectomy. (Conventional microdiscectomy is now considered common surgical treatment for lumbar disc herniation).

Several minimally invasive surgical approaches have been introduced for the surgical management of symptomatic lumbar disc herniation. The effectiveness of these procedures should be compared with lumbar microdiscectomy. Systematic reviews comparing specific types of minimally invasive lumbar surgery for management of lumbar disc herniation and lumbar radiculopathy, but did not yield conclusive results due to a lack of evidence. In this paper, we perform a systematic review of the literature and draw conclusions about safety and efficacy of minimally invasive discectomy compared to standard microdiscectomy.

**Keywords:**

Minimally invasive discectomy, Microdiscectomy, Disk herniation


					Volume 4, Suppl. 1 Nov 2012
Publisher: Kermanshah University of Medical Sciences
URL: http://www.jivresearch.org
Copyright:
Congress of Iranian Neurosurgeons, 14-16 November 2012, Kermanshah, Iran
Paper No. 61
Minimally invasive discectomy versus microdiscectomy/
discectomy for symptomatic lumbar disc herniation
Vafa Rahimi-Movaghar a,b
, Mohammad Rasouli c
, Farhad Shokraneh d
, Maziyar Moradi-lakeh e
,
Alex Vakaro f
, Mohsen Sadeghi-Naini e,*
a
Deparment of Neurosurgery - Sina Hospital, Tehran University of Medical Sciences, Tehran, Iran.
b
Research Centre for Neural Repair, University of Tehran, Tehran, Iran.
c
Rothman Institute of Orthopaedics, Thomas Jefferson University, Philadelphia, PA, US.
d
Medical Librarianship and Information Sciences, Tabriz University of Medical Sciences, Tabriz, Iran.
e
Tehran University of Medical Sciences, Tehran, Iran.
f
Department of Orthopaedic Surgery and Neurosurgery,Thomas Jefferson University Hospital, The Rothman Institute.
Abstract:
Background: Lumbar discectomy is a surgery to remove all or part of a disc cushion that helps protect the spinal
column. These cushions, called disks, separate the spinal vertebrae/bones. When one of the disks herniates (moves
out of place) in patients with protruded disc, the soft gel inside pushes through the wall of the disk. The disk may then
place pressure on the spinal cord and nerves that are coming out of the spinal column.
The lumbar discectomy procedure remained basically unchanged until the operating microscope enhanced the
visualization of the operative field in 1978. This new operation was recognized as lumbar microdiscectomy because
it was performed through a smaller incision, with less dissection than standard open lumbar discectomy.
Microdiscectomy is regarded generally as a technical modification of standard discectomy, rather than a separate
procedure. In a systematic review by Gibson and Waddell, results of microdiscectomy for treatment of lumbar disc
prolapse was "broadly comparable" to the standard open lumbar discectomy. (Conventional microdiscectomy is now
considered common surgical treatment for lumbar disc herniation).
Several minimally invasive surgical approaches have been introduced for the surgical management of symptomatic
lumbar disc herniation. The effectiveness of these procedures should be compared with lumbar microdiscectomy.
Systematic reviews comparing specific types of minimally invasive lumbar surgery for management of lumbar disc
herniation and lumbar radiculopathy, but did not yield conclusive results due to a lack of evidence. In this paper, we
perform a systematic review of the literature and draw conclusions about safety and efficacy of minimally invasive
discectomy compared to standard microdiscectomy.
Keywords:
Minimally invasive discectomy, Microdiscectomy, Disk herniation
* Corresponding Author at:
Mohsen Sadeghi-Naini: Tehran University of Medical Sciences, Tehran, Iran. Tel: 09124934734, Email: dr.msadeghi@yahoo.com, (Sadeghi-Naini M.).

				

